# 

**Published:** 1822-06-01

**Authors:** 


### Medico-Chirurgical Review,

AND

### JOURNAL

OF

### MEDICAL SCIENCE.

(Quarterly.)

EXHIBITING A COMPREHENSIVE ANALYTICAL RECORD
OF PROGRESSIVE MEDICINE AND SURGERY;

*EQUALLY ADAPTED TO ALL RANKS OF THE PROFESSION.*

CONDUCTED BY

ASSOCIATED PHYSICIANS AND SURGEONS;

AND SUPERINTENDED BY

JAMES JOHNSON, M. D.

MEMBER OF THE ROYAL COLLEGE OF PHYSICIANS OF LONDON.

(Analytical Series.)

S. G. O. 26569 LIBRARY

VOLUME III. for 1822-3.

Nec tibi quid liceat sed quid fecisse decebit

Occurrat mentemque domat respectus honesti. CLAUD.

London:

Printed by G. HAYDEN, Little College Street, Westminster,
FOR THE EDITOR,
No. 58, Spring Gardens,
[Where Books for Review and Critical Communications, &c. *(post paid)* are to be directed.]

Published by BURGESS & HILL, Windmill Street, Haymarket; BALDWIN, CRADDOCK, & JOY, Pater-Noster Row; PARBURY, & Co. Leadenhall Street; HIGHLEY, and T. & G. UNDERWOOD, Fleet Street; CALLOW and WILSON, Princes Street, Soho; COX and SON, Borough; JACKSON, Borough; ANDERSON, West Smithfield; JOHN COX, Berners Street, Oxford Street; ADAM BLACK, Edinburgh; RICHARD GRIFFIN & Co. Glasgow; HODGES and M'ARTHUR, Dublin; Messrs. TREUTTLE and WURTZ, Paris and Strasburgh.

1823.

W1
ME750

☞ As misconceptions are constantly taking place respecting the mode of procuring this Journal, we have again to state, that every Subscriber is to procure it through the medium of his own bookseller, as we never send the work to subscribers ourselves.

We have, also, to inform our professional brethren, that we cannot give insertion to any Reports of Dispensaries, Regulations of Colleges or Universities, Prospectuses of Lectures, &c. excepting as advertisements, and to be paid for accordingly.

The extra-limites department is open, as usual, for authors to defend themselves against criticisms in this and all other journals, such defences being at the expence of the authors themselves.

### XII.

### BIBLIOGRAPHICAL RECORD;

OR

*Works received for Review within the Quarter.*

1. Ad acutæ et chroniæ Splenitidis, in humilibus præsertim Italiæ locis, consideratæ, eidemque succedentium Morborum Historias Animadversiones. Auctore, STANISLAO GROTTANELLI, Philosophiæ, Medecinæ, et Chirurgiæ Doctore, &c. Florentini, 1822.

---

2. The Pathology of Fever; being the Subject of the Gulstonian Lecture, lately delivered at the Royal College of Physicians. By J. R. PARK, M.D. Fellow of the Royal College of Physicians. Octavo, pp. 161. London, 1822.

---

3. Further Observations on Strictures of the Rectum; with Remarks on the Opinions of some late Writers relative to the Situation of the Disease; and also, on Spasmodic Constriction of the Sphincter Ani; with, a Translation of Part of M. Boyer's valuable Paper on that Complaint; accompanied with several Cases, and an Engraving. By W. WHITE, Member of the Royal College of Surgeons, London; and one of the Surgeons to the Bath Infirmary and Dispensary. Octavo, pp. 105, with an Engraving. London, 1822.

---

4. Travels into the Baga and Sooso Countries, during the year 1821. By PETER M'LACHLAN, Esq. Assistant Staff-Surgeon, and one of the Colonial Surgeons of Sierra Leone. Octavo, sewed. Sierra Leone, 1821.

☞ *We shall be happy to see Mr. M'Lachlan's promised work on the medicine and medical topography of the countries through which he passed, with so much credit to his personal courage and scientific acquirements.*

---

5. A Manual of Anatomy; containing Rules for Displaying the Structure of the Body, so as to exhibit the elementary Views of Anatomy, and their Application to Pathology and Surgery: to which are added, Observations on the Art of Making Anatomical Preparations. By JOHN SHAW; being an Outline of the Demonstrations delivered by him, to the Students in the School of Great Windmill Street. *Second Edition*, 8vo. pp. 444, with Plates. London, 1822.

☞ *After what we have said of the first edition of this work in our Seventh Number, it is unnecessary to do more than announce a new and improved edition at the present time.*

---

6. An Address, delivered in the Guildhall at Plymouth, on the 6th day of December, 1821, at the First general Meeting of the Subscribers to the Plymouth Eye Dispensary. By JOHN BUTTER, M.D. F.L.S. Octavo. Plymouth, 1822.

☞ *The general utility of such institutions is here well advocated, and the objections to them over-ruled.*

7. An Account of the new Institution in Edinburgh, for the Application of Vapour, Mineral Water Baths, &c. No. 8, Broughton Street, (Edinburgh,) with the History of the successful Cases that have occurred there. Octavo, pp. 71. Edinburgh, 1822.

☞ *We are glad to observe that so very useful an institution is patronized in the Northern Metropolis. The proprietor is evidently taking the surest means of obtaining ultimate and permanent success. The application of the baths is under the guidance of medical science, and no system of popular delusion, appears to enter into the economy of the institution. We hope the Faculty will strenuously support it.*

---

8. Observations on the Influence of Habits and Manners, National and Domestic, upon the Health and Organization of the Human Race;—and particularly on the Effect of that Influence as it relates to the present State of English Females, in the higher and middle Classes of Life. By Ralph Palin, M.D. One Volume, 8vo. pp. 297. London, 1822.

---

9. The Medical Practitioner's Pocket Companion; or a Key to the Knowledge of Diseases, and of the Appearances that denote Recovery or Danger: being an Alphabetical Arrangement of Symptoms, with their various Indications. Duodecimo, pp. 32, small print. London, 1822.

☞ *This little work has the merit of novelty, and may not be devoid of utility, when bound up with Thompson's Conspectus, with which it is printed uniform. We cannot do better than give a single specimen, which will convey a very good idea of the nature of the work. D stands for "denotes," and P, "prognosis."*

"Fæces, chronic discharge of liquid, indigested aliment resembling chyle.—D. *Cœliaca passio.*

" ——— frequent discharge of, loose and watery, sometimes mixed with blood.—D. *Diarrhœa.* (Without fever generally, but sometimes with more or less fever of the inflammatory kind.)

" ——— frequent mucous or bloody stools, accompanied with much griping, and followed by a tenesmus, the alvine fæces being for the most part retained; fleshy or sebaceous lumps are sometimes discharged. (Attended with fever of the nervous and putrid kind, and considerable prostration of strength.)—D. *Dysentery.*

" ——— highly acrid aad obstinate discharge of, resembling dysentery, and corroding all the parts they touch; attended with frequent convulsions, and fixed pains.—D. *Cancer in the intestines.*

" ——— purulent, preceded by throbbing pain in some part of the abdomen, with shivering and fever, and exacerbation of the symptoms in the evening.—D. *Abscess in the intestines.*

" ——— accompanied with discharge of pieces of membrane.—D. *Abscess in the intestines.*

" ——— accompanied with pus.—D (Sometimes) *Abscess of the Mesentery.*

" ———with an offensive, putrid smell, the food passing crude and undigested.—D. *Diseased Liver.*

" ——— pale and whitish —D. *Stones in the gall-duct. Jaundice.*

" ——— blackish, and very offensive, often passing off insensibly.—D. *Putrid fever.*

" ——— involuntary discharge of.—D. *Compression of the Brain.*—D. *Great danger* (in various diseases).

"Fæces, voided unconsciously (in hydrocephalus).—P. *Approaching death.*

" ——— (in jaundice), whiteness of, changing to a more natural colour. —P. *Favourable.*

" ——— (in splenalgia), discharge of black bilious.—P. *Favourable.*

" ——— (in cholera morbus), continual urging to discharge.—P. *Fatal.*

" ——— (in fever), scybala brought off with little straining or colic.—P. *Favourable.*

" ——————— liquid, frothy, watery, with little colour or smell.—P. *A tedious disease.*

" ——————— a free and copious discharge of, highly fetid and bilious (in the beginning of the disease).—P. *Rather favourable.*

" ——— (in putrid fever), ichorous and fetid.—P. *Highly dangerous.*

" ——— (in bilious fever), cadaverous.—P. *Approaching death.*

" ——— (in hectics), highly liquid and offensive.—P. *Extreme danger.*

" ——— small, black, pitch-like.—P. *Danger.*

---

10. American Medical Recorder, No. 17, for January 1822.

☞ *We are most happy to learn, that the circulation of this respectable transatlantic cotemporary already amounts to* 1750—*no mean circulation for a medical journal in any country—and not now to be attained by ordinary merit.*

---

11. Lettera di Giacomo Clark, M.D. della Universita di Edimburgo, Al. Ch. Sig. Professore Tommasini, uno Dei 40 della Societa Italiana ec. Intorno Alle sue Osservazioni sulla Scuola Medico-Clinica di Edimburgo, contenute nel suo Discorso del Metodo di Curare dell' insegmento Medico-Clinico, &c. Observati in Inghilterra, prononciato nella Clinica Medica della Pontifica Universita de Bologna, il 26, Marzo 1821. Roma, 1822.

---

12. Essays Physiological and Practical. By James Carson, M.D. Physician in Liverpool. Octavo, sewed, pp. 65. 1822.

☞ *The first two Essays are republications, and have been noticed before in this Journal. The third Essay is on Lesion of the Lungs, and contains a Proposition for the Cure of Phthisis, not before suggested by any author. It will be found in another part of the Journal.*

---

13. A Pharmaceutical Guide; in two Parts. Part I. A Latin Grammar, in which all the Rules are illustrated by Examples, taken from the London Pharmacopœia. Part II. An Interlineary Translation of such Formulæ in the London Pharmacopœia, as have been found difficult to be comprehended by some young Medical Students. To which is affixed, a Vocabulary of Words most frequently employed in Prescriptions; with Examples of their Uses. By the Author of the Student's Manual. One small Volume, duodecimo, pp. 92. London, 1822.

---

14. A Case of Transverse Fracture of the Patella, in which, perfect Osseous Union was procured; with Observations. By George Fielding, Member of the Royal College of Surgeons in Edinburgh; one of the Surgeons to the Infirmary, to the Lying-in-Charity, and to the Female Penitentiary in Hull. Octavo, sewed, p. 10. 1822.

15. The new Medico-Chirurgical Pharmacopœia; being a Selection of modern Formulæ, from the private and hospital Practice of the most eminent Members of the Profession, in Europe and America: for the Use of Surgeons and Surgeon-Apothecaries. By a Member of the Colleges of Surgeons, of London and Edinburgh. Small Octavo, pp. 148. Price, 5*s.* 6*d.* boards. London, 1822.

---

16. Bulletins de la Societé d'Emulation de Paris, et Tablettes Medico-Chirurgicales. Redigée par M. M. Bricheteau et Vilerme. January, February, and March, 1822.

☞ *In exchange for Medico-Chirurgical Review.*

---

17. Letters to the Honorable the Managers of the Royal Infirmary, occasioned by an Extraordinary Resolution they have lately entered into. By Robert Liston, Surgeon, &c.

"Non metuunt leges, sed cedit viribus æquum."

☞ *Page* 207 *of Periscope.*

---

18. A System of Surgical Anatomy. Part I. On the Structure of the Groin, Pelvis, and Perineum, as connected with Inguinal and Femoral Hernia; Tying the Iliac Arteries; and the Operation of Lithotomy. Illustrated by Nine copper-plate Engravings. By William Anderson, Licentiate of the Royal College of Surgeons in Edinburgh, and Lecturer on Surgical Anatomy in New York. Quarto, pp. 200, with Nine coloured Plates. New York, 1822.

☞ *We return the author our thanks for this volume, and have put it into the hands of one of our surgical conductors for early notice.*

---

19. Morgagni's Epistles on the Seat and Causes of Diseases; abridged, with copious Notes; by William Cooke, Member of the Royal College of Surgeons, and one of the Secretaries to the Hunterian Society. In 2 Vols. thick octavo, pp. 500 each volume. Longman and Co. 1822. (First volume received.)

---

20. Some Observations on the present Practice of Inoculating Children for the Small-pox, in the Neighbourhood of Chichester and Bognor. By John Conolly, M.D. Member, and late President, of the Royal Medical Society of Edinburgh, &c. Octavo, sewed, pp. 29. 1822.

☞ *A well written appeal in favour of vaccination.*

---

21. An Enquiry into the comparative Forces of the Extensor and Flexor Muscles, connected with the Joints of the Human Body. By Julius Jeffreys, Member of the Royal College of Surgeons in London. Octavo, pp. 51. London, 1822.

---

☞ Subscribers are those who procure the Journal through the medium of their own Booksellers, to whom they are to pay for the same, as it is quarterly procured, there being no Subscription necessary in advance.

*Erratum in last No.*

No. 8, p. 907, (Dr. Emerson's Prospectus,) for "forms and *preparations*," read "forms and *properties*," of vegetables.

Aked, Mr. R. Surgeon, Ha fax.
Baron, ——, Esq. Surgeon, New Kent Road
Batt, Benjamin, Esq. Clifton
Bell, Thomas, Esq. Deeping, Lincolnshire
Blaken, Mr. Brighton
Borthwick, G. A. (M. D. F. R. S. Ed.) George Street, Edinburgh
Callow, Mr. Surgeon, Thornton, Yorkshire
Carrol, Mr. Wm. Surgeon, R. N. Walworth Road
Churchill, Mr. J. M. Princes St. Leicester Square
Conolly, Dr. John, Chichester
Constantia, Dr.
Cooke, Dr. J. Henry, Landport Terrace, Portsmouth
Correy, Mr. Student, Guy's Hosp.
Coxon, Mr. (Student) London
Desmond, John, M. D. Bolton, near Lancaster.
Diel, Dr. Three Towns, N. America
Dymond, John, M. D. Bolton, near Lancaster
Dymond, Robert, M.D. Member Extraordinary of the Royal Medical Society of Edinburgh, Florence
Editors of "Bulletins de la Societé Medicale d'Emulation," in exchange
Elliot, Mr. Surgeon, Lee
Evans, Mr. Surgeon, Brixton
Farran, William, M. D. Barnsley, Yorkshire
Fraser, Mr. (by mistake inserted Dr.) Doughty Street
Freer, Mr. Surgeon, Leicester
Gilpin, Bernard, Esq. Memb. of Royal Coll. Surg. of London Ulverstone, Lancashire
Graves, Mr. W. Surgeon, Louth
Greenwich and Deptford Medical Book Societies
Hewlet, Mr. Student, Guy's Hosp.
Huddersfield Medical Book Society
Jackson Mr. Surgeon, Helperby
Jessop, Edward, M. D. Assistant Surgeon, Madras Establishment
Jordan and Stratton, Messrs.
Kyd, Lieut.-General, Albemarle Street
Leicester Medical Book Society
Little, Mr. Surgeon, ———, near Plymouth
Lloyd, Mr. Williams, Surg. Derby
Macdonnel, Mr. Surgeon, Gerrard Street, Soho
Mackbeth, James, Esq. Ayreshire
Mangles, Mr. Surg. York.
Marchant, Mr. Surg. North Cray
Marriott, John, Jun. Esq. Kibworth
Martin, Mr. Robert, Surg. Hon. East India Company's Service, Bombay
Nason, E. Esq. Member of Royal College of Surgeons, London, Nuneaton, Warwickshire
Newstead, Mr. Surgeon, Bubwith
Ormsby, Dr.
Osborne, Mr. Surgeon, Hanslope, Bucks.
Owen, Mr. Oxford Street
Patton, Mr. David, Surgeon, His Majesty's Cutter, Vigilant
Pratt, Mr. Surg. Dorset Street, Portman Square
Quin, Dr. Resident Physician, Naples
Robert, Dr. Beaumaris, Wales
Robertson, Dr. Canada
Smith, Dr. John, Maidstone
Smith, Dr. Prichard, (place not stated at Callow's)
Stephenson, Mr. William, Surg. Grenada
Thomson, Dr. George, H. M. S. Liffey, to East Indies
Thurston, Mr. John, Surgeon, Norwich
Trueman, Mr. Great Russel St.
Tullock, Mr. Benj. Membeo of Royal College of Surgeons, Newcastle-upon-Tyne
Verner, Ed. M.D. (Dublin) Memb. of the Royal College of Surg. London, and late Assist. Surg. Royal Horse Artillery
Walhound, Dr. S. Hereford
Webster, Mr. Philadelph. (through Cox and Co.)
Wise, John, Esq. Banbury
Withering, Mr. Rob. M.C.S. Lond. Surg. Royal Navy, Tenby, S. Wales
Woodthorpe, Mr. Surgeon, R. N.
Woodward, Mr. George, Surgeon
Woolcot, Mr. Surg. Hon. Comp. Service
Woolstenholme, Mr. Surgeon, Holywell

